# Effect of dietary *Arthrospira platensis* phycocyanin on broiler chicken growth performance, physiological status, fatty and amino acid profiles

**DOI:** 10.14202/vetworld.2024.1098-1107

**Published:** 2024-05-17

**Authors:** Niamat M. El-Abd, Ragaa A. Hamouds, Amna A. Saddiq, Turki M. Al-Shaikh, Tibra J. Khusaifan, Ghada Abou-El-Souod

**Affiliations:** 1 Sustainable Development of Environment and its Projects Management, Environmental Studies and Research Institute, University of Sadat City, Sadat City 32897, Egypt; 2 Department of Biology, College of Science and Arts at Khulis, University of Jeddah, Jeddah 21959, Saudi Arabia; 3 Genetic Engineering and Biotechnology Research Institute, University of Sadat City, Sadat City 32897, Egypt; 4 College of Sciences, University of Jeddah, Jeddah, Saudi Arabia; 5 College of Art and Design, University of Jeddah, Jeddah, Saudi Arabia; 6 Department of Botany and Microbiology, Faculty of Science, Menoufia University, Shibin Al Kawm, Egypt

**Keywords:** Antioxidant, fatty acid, Phycocyanin, poultry, protein, *Spirulina*

## Abstract

**Background and Aim:**

Natural antioxidants are crucial for preserving and enhancing the health, survival, reproduction, and reproductive function of poultry. Phycocyanin (PC) is a natural blue food colorant with various health benefits. The aim of this study was to extract *Arthrospira platensis* phycocyanin (ApPC) from *A. platensis* using simple and economical methods and investigate the impact of phytocyanin supplementation on the performance and fatty and amino acid profiles of broiler chicks.

**Materials and Methods:**

PC was extracted from *A. platensis* by freezing and thawing, and optimization conditions such as pH and temperature were applied during storage periods. A total of 270 1-week-old Ross breed broiler chicks were randomly assigned to the following three treatment groups: basal diet supplemented with 0 mg of PC/kg diet (control), basal diet supplemented with 1 g PC/kg diet (T1), and basal diet supplemented with 2 g PC/kg (T2). In a completely randomized design, three cage replicates (30 birds each) were assigned to each of the three groups. The dietary effects of ApPC on growth performance (body weight gain [BWG], body weight [BW], feed intake, feed conversion ratio, serum constituents, and antioxidant indices) in broiler chickens, free amino acids, and fatty acids in muscles were evaluated.

**Results:**

Total BWG and BW increased without a significant effect on the total feed consumption. Serum levels of total proteins and albumin increased with increasing ApPC supplementation. In addition, globulin levels significantly increased. There was a significant decrease in serum total cholesterol levels among the treatments. The activity of antioxidant enzymes (superoxide dismutase, catalase, glutathione, and total antioxidant capacity) is significantly increased. In contrast, an increase in ApPC caused a significant decrease in malondialdehyde. The content and quantity of fatty acids and amino acids in the meat of broiler chicks supplemented with PC varies.

**Conclusion:**

The addition of PC to broiler chicken diets enhances antioxidant activities, BW, BWG, and meets quality requirements.

## Introduction

The use of natural ingredients instead of antibiotics, growth hormones, or other chemicals is a current trend in poultry feeding. Natural components are currently utilized instead of growth hormones and antibiotics in poultry nutrition [1]. Naturally occurring antioxidants are recommended in broiler production because they promote health by reducing the generation of reactive oxygen species (ROS) and the ensuing oxidative stress [2]. Safari *et al*. [3] reported that phycocyanin (PC) is a natural antibacterial product that is harmless.

Microalgae are photosynthetic organisms that generate algal biomass from sunlight, water, and CO_2_. Certain species are abundant in proteins, minerals, carbohydrates, and other vital substances [4]. Microalgae are a high-nutrient natural feed source that could be a potentially useful ingredient in chicken diets [5]. *Arthrospira platensis* is an aquatic, filamentous cyanobacterium that is often classified as a blue/green microalga. The common name of its commercialized biomass is *Spirulina*. *A. platensis* is the most cultivated microorganism worldwide [6]. *Spirulina platensis* is a microalga with biological activity that is used to produce nutritional supplements rich in vitamins, proteins, essential fatty acids, and minerals [7]. Arthrospira is a blue-green photosynthetic filamentous alga that contains phycobiliprotein pigments (PC, phycoerythrin, and allophycocyanin), 15%–25% carbohydrates, 18% essential fatty acids, 55%–70% proteins, vitamins, and minerals. It also contains high levels of gamma-linoleic acid and phenolic acids [8]. The protein content of *A. platensis* ranges from 50% to 70% [9].

Numerous studies using laboratory animals have confirmed the benefits of *Spirulina*, including anti-inflammatory or immunostimulating features, hypolipidemic activity, and antioxidant properties [10]. The addition of *S. platensis* as a feed supplement positively impacts productivity and immunity in broiler chickens, improving humoral and cellular immune responses and lymphoid organ development [11].

Dietary *Spirulina* supplementation of chickens under heat stress conditions could reduce the adverse effects of chronic heat stress on growth performance and immunity [12]. Dietary inclusion of *S. platensis* can potentially mitigate heat stress in broilers [13]. Fernandes *et al*. [14] discovered that only a few of the health advantages of *A. platensis* are growth characteristics with hypolipidemic, antioxidative, anti-inflammatory qualities, and immune stimulation. *S. platensis* and organic selenium in the diet improved immunoglobulin levels, glutathione peroxidase (GPx), superoxide dismutase (SOD), and productive performance while lowering blood levels of malondialdehyde (MDA) [15]. Microalgae are a high-nutrient natural feed source and could be a potentially useful ingredient in chicken diets [16].

A blue photosynthetic pigment known as PC is found in some red algae and cyanobacteria. It is water-soluble in the membrane of the cytoplasm [17]. PC can be extracted from Arthrospira using both inorganic and organic solvents, freezing, thawing, homogenizing enzymes, and ultrasonography [18]. Xalxo *et al*. [19] discovered that PC has antioxidant, hepatoprotective, anticancer, radical scavenging, anti-inflammatory, anti-arthritic, and immune-boosting properties. Due to their ability to scavenge alkyl, hydroxyl, and peroxyl radicals, as well as their hydroxyl and aromatic substituent structures, PC is credited with antioxidant activity.

Therefore, this study aimed to assess the impact of *A. platensis* phycocyanin (ApPC) levels as a food additive on the antioxidant capacity, growth performance, and blood biochemical components of broiler chicks. PC extraction by simple methods from *A. platensis* was also studied to determine its stability during storage periods at different temperatures and pH conditions.

## Materials and Methods

### Ethical approval

All experimental procedures were performed in accordance with the ethical guidelines of Genetic Engineering and Biotechnology Research Institute, University of Sadat City, Egypt, under approval number SREC 014223B50015.

### Study period and location

This study was conducted during December 2022 and January 2023 at the private poultry field in Kafr El-Sheikh Governorate.

### PC extractions

*A. platensis* alga was purchased from the National Research Center, Egypt, and dried at 60°C to a consistent weight. The C-PC extraction was performed using freeze/thaw methods in phosphate buffer at pH 5, 7, and 8. The freeze/thaw method was repeated 3 times every 24 h [20].

PC stability under various storage conditions.

The stability of PC was determined under different storage conditions (temperature, 4, 25, and 40°C; pH, 5, 7, and 8) for 3, 5, and 7 days.

### Determination of PC content

C-PC concentrations were measured at optical densities of 620 and 652 nm and calculated using Equation 1 [21].

C-PC = (A620–0.474 A652)/5.34)

Yield was measured as C-PC milligrams/dry biomass.

### Experimental design

This investigation was conducted at the Environmental Studies and Research Institute, University of Sadat City for 35 days. A total of 270 1-week-old Ross breed broiler chicks were selected at random and assigned to three experimental groups: a basal diet with no supplementation (control, 0 g of PC/kg diet), T1 1 g PC/kg diet, and T2 2 g PC/kg diet. Treatments were assigned to three cage replicates (30 chicks each) for each of the three groups in a completely randomized design. To comply with all recommendations, diets have been modified to achieve isocaloric and isonitrogenous levels. The trail diet was designed to meet the dietary needs listed in Table-1 as recommended by the National Research Council [16, 22].

**Table 1:** Composition and calculated analysis of the experimental diet.

Ingredients	Starter diets	Grower diets
Yellow corn (9%)	51.73	58.80
Soybean meal (44%)	40.01	33.02
Vegetable oil	5	4.61
Di calcium phosphate	0.24	0.24
Vitamin and mineral mixture^1^	0.50	0.50
Sodium chloride	0.30	0.30
Limestone	2.0	2.31
DL-Methionine^2^	0.22	0.22
Total	100	100
Calculated analysis (air dry basis)^3^		
Crude protein, %	22.00	20.02
ME, kcal/kg diet	3110	3100
C/P ratio	140	153
Available phosphorous, %	0.40	0.40

1 Vitamins and minerals mixture at 0.30% of the diet supplies the following/kg of the diet: Vit. A, 12,000 IU; Vit. D_3_, 2500 IU; Vit. E, 10 mg; Vit. K_3_, 3 mg; Vit B_1_, 1 mg; Vit. B_2_, 4 mg; Pantothenic acid, 10 mg; Nicotinic acid, 20 mg; Folic acid, 1 mg; Biotin, 0.05 mg; Niacin, 40 mg; Vit. B_6_, 3 mg; Vit. B_12_, 0.02 mg; Choline chloride, 400 mg; Mn, 62 mg; Fe, 44 mg; Zn, 56 mg; I, 1 mg; Cu, 5 mg and Se, 0.01 mg.

2 DL - Methionine: 98% feed grade (98% Methionine).

3 Calculated according to National Research Council [16], ME=Metabolizable energy

### Measurements and techniques

The following criteria were computed and/or calculated for the feeding trial: body weight (BW), feed conversion ratio (FCR), feed intake (FI), and BW gain (BWG).

At the end of the 35-day trial, as soon as the birds were slaughtered, all birds handling procedures, sample collection, and disposal were performed according to the Genetic Engineering and Biotechnology Research Institute, University of Sadat City, Egypt, under approval number SREC 014223B50015. Blood samples were collected and stored in heparinized tubes. Plasma total protein (TP, g/dL), globulin (GL, g/dL), albumin (Alb, g/dL), cholesterol (CHO) (mg/dL), aspartate aminotransferase (AST) (u/L), and alanine aminotransferase (ALT) (u/L) were identified using a spectrophotometer on an individual basis according to the manufacturer’s instructions.

### Antioxidant enzyme activity in liver tissues

In the control and experimental groups, the livers were chopped, weighed, and minced into small pieces (approximately 0.5 g of each). The livers were placed in Eppendorf tubes (Sigma-Aldrich^®^ Darmstadt, Germany) and stored at –80°C until biochemical analysis.

### Preparation of the liver homogenates

To eliminate any red blood cells and clots, liver tissues were perfused with phosphate-buffered saline (PBS; pH 7.4) solution containing 0.16 mg/mL heparin before dissection. After dividing the liver tissues into appropriate sections, 0.5 g of each was homogenized using a tissue homogenizer in 5 mL of 10% (w/v) cold PBS, which is equivalent to 50 mM potassium phosphate, pH 7.5, and the resulting supernatant was centrifuged at 6708 × *g* for 20 min at 4°C. Oxidative stress and antioxidant biomarkers, such as L-MDA, reduced glutathione (GSH), SOD, and catalase (CAT), were measured using the techniques outlined by Esterbauer *et al*. [23].

### Preparation and determination of free amino acid samples

For free amino acid analysis, approximately 240 mg of tissue was homogenized in 3 mL of a mixed solvent (acetonitrile: water 1:1). Subsequently, the suspensions were shaken for 60 min, centrifuged at 12225 × *g* for 10 min, and the supernatants were collected [24]. High-performance liquid chromatography (HPLC) was used to assess the amino acid content [25]. Agilent HPLC (USA) 1260 Infinity was an HPLC system that included a quaternary pump, an ultraviolet (UV) detector, and a thermostatted column compartment (TCC) sampler; a unit controlled by a computer was utilized. The mobile phase gradient between the two solvents varied over a 20 min run duration, from 5% to 70% (w/v) and solvent A ammonium acetate buffer at 50 mM, pH 6.5. Solvent B: acetonitrile: 50:50, 100 mM ammonium acetate, 2 mL/min of flow. UV light was detected at 254 nm, and the column was maintained at 50°C during the chromatography process. The RP-18 column (250 × 4 mm, 5 μm i.d.) was shielded with a guard column made of the same material (Merck, USA) during the separation procedure.

Glutamic acid, aspartic acid, glycine, serine, histamine, taurine, arginine, valine, methionine, proline, tyrosine, threonine, leucine, isoleucine, lysine, and cysteine were obtained from Sigma (St. Louis, MO, USA).

### Analyzing meat fatty acid content

Approximately 2 mL of n-hexane was added to 0.1 g of tissue and shaken at 50°C for 30 min followed by 3 mL of potassium hydroxide methanol solution (0.4 mol/L). The samples were then stirred for 30 min at 50°C at 200 rpm. Subsequently, 1 mL of water and 2 mL of n-hexane were added, and the mixture was blended. The mixture was allowed to rest for stratification. Fatty acids were detected using gas chromatography (GC)-mass spectrometry [25]. Gas chromatographic system (TraceGC model K07332, Hermo Finnigan, Thermo Quest, Milan, Italy) and the chromatographic conditions were as follows. The carrier was N2, the flow rate was 1 mL/min, the oven was set to 70°C for 0.5 min, then increased by 30°C/min to 180°C for 10 min, and by 5°C/min to 225°C for 15 min. The intake and detector temperatures were 250°C and 1 μL with split 1/20. Fatty acid methyl ester (FAME) retention times and elusion order were identified using Supelco (Milan, Italy), as reference standards.

### Statistical analysis

The obtained findings were statistically analyzed using one-way analysis of variance (ANOVA) (the Statistical Package for the Social Sciences, version 22 for Windows) (SPSS Inc., Chicago IL USA). Duncan’s Multiple Range [26], Duncan’s multiple range test was used to differentiate between significant means at p < 0.05.

## Results and Discussion

### Extract of PC from *A. platensis*, yield, and stability

**Figure-1:**
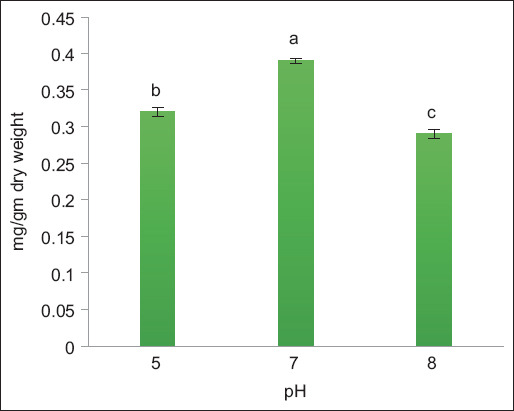
Effect of pH on phycocyanin extracted from *Arthrospira platensis* as (mg/g) dry weight at 25°C (bars: errors bar, different letters are significant values).

Pigment contents of *A. platensis* at pH 5, 7, and 8 on day 3 after extraction are presented in Figure-1. The maximum PC content was observed at pH 7 (0.39 mg/g dry weight), followed by pH 5, and a low content was observed at pH 8. The PC content was extracted from *A. platensis* using the freeze/thaw technique and recorded at 4.88 mg/g dry weight [27]. The proportion of PC extracted from *A. platensis* was 4.665–5.465 mg/g biomass using water in all extraction procedures compared to other solvents [20].

**Figure-2:**
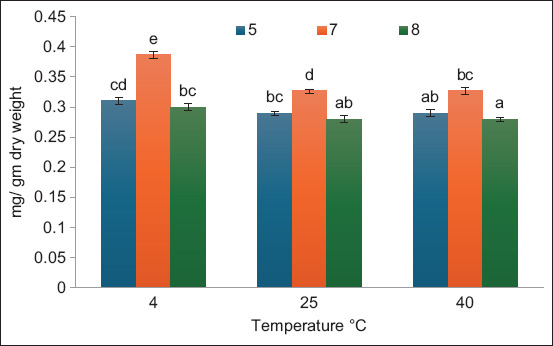
Effect of temperature and pH on phycocyanin extracted from *Arthrospira platensis* as mg/g dry weight at 3 days storage periods (bars: errors bar, different letters are significant values).

**Figure-3:**
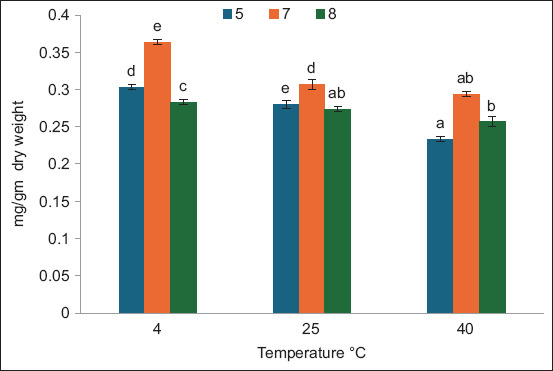
Effect of temperature and pH on phycocyanin extracted from *Arthrospira platensis* as mg/g dry weight of at 10 days storage periods (bars: errors bar, different letters are significant values).

**Figure-4:**
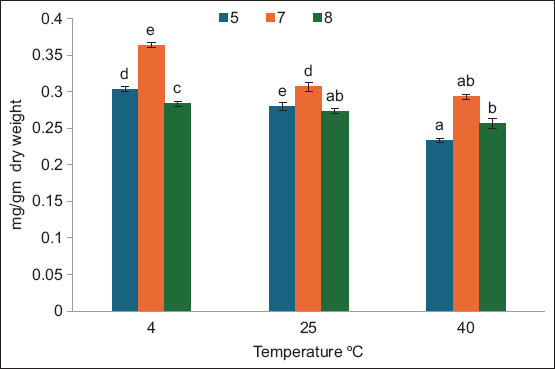
Effect of temperature and pH on phycocyanin extracted from *Arthrospira platensis* as mg/g dry weight at 17 days storage periods (bars: errors bar, different letters are significant values).

Figure-2 shows the effects of temperature and pH during the storage period 3 days after extraction. There were significant effects among the variables (PC yield as mg/g dry weight). The highest values were observed at 4°C and pH 7 followed by pH 5 at the same temperature. The PC contents were observed at 40°C, pH 8 and 5, whereas there was no significant difference between the PC contents at 40°C with pH 5 and at 25°C with pH 8. The results in Figure-3 indicate how pH and temperature affect the PC yield as mg/g dry weight of *A. platensis* after 10 days of storage. The best results of stability were observed at pH 7, followed by pH 5 at different temperatures. Low temperature and pH 7 were the most effective factors that maintained low degradation of PC compared with other tested temperature and pH values. Figure-4 demonstrates the same results obtained, showing that pH 7 and low temperature maintained PC stability for 17 days of storage. Overall, pH 7 maintained PC stability for 3, 10, and 17 days of storage. For PC *A. platensis* stability, storage conditions of pH 7 and –20°C were efficient [20]. pH 9 strongly reduces the stability of PC at 49.9°C [28]. The ideal parameters for maintaining the long-term stability of food-grade PC extracted from *A. platensis* are low temperature, darkness, and pH between 5.0 and 6.0 [29]. The conditions that affect the stability of phytocyanin differ according to the type of algae. PC extracted from *Nostoc sphaeroides* remained stable at 50°C and pH 6–8 [30].

### Relationships among factors

Table-S1 shows the relationship between pH, temperature, and storage period of PC yield (mg/g dry weight) of *A. platensis*. The results indicate that there are significant effects among pH, temperature, and storage (p ≤ 0.000) on PC stability in *A. platensis*; therefore, it is necessary to determine which factors affect PC stability. Table-S2 shows a two-way ANOVA of the relationships between pH and temperature in each storage period for PC stability in *A. platensis*. The results showed that pH and temperature had significant effects on PC stability during storage periods (3, 10, and 17 days). The adjusted R2 was >0.9, indicating that the type of model was effective and could accurately predict the response [31].

**Table-S1:** Three ways ANOVA of the relations among pH, temperature, and storage periods in related to Phycocyanin of *A. platensis* yield as mg/g dry weight.

Tests of Between-Subjects Effects						

Dependent Variable: dryweight						

Source	Type III Sum of Squares	df	Mean Square	F	p-value	Partial Eta Squared
Corrected Model	0.117^a^	26	0.004	61.714	0.001	0.967
Intercept	6.599	1	6.599	9.060E4	0.001	0.999
Storage period	0.020	2	0.010	134.305	0.001	0.833
pH	0.044	2	0.022	299.458	0.001	0.917
Temp	0.044	2	0.022	302.305	0.001	0.918
Storage period *pH	0.001	4	0.000	1.822	0.138	0.119
Storage period *Temp	0.000	4	6.235E-5	0.856	0.496	0.060
pH *Temp	0.006	4	0.001	20.407	0.001	0.602
Storage period *pH *Temp	0.003	8	0.000	5.013	0.001	0.426
Error	0.004	54	7.284E-5			
Total	6.720	81				
Corrected Total	0.121	80				

a. R Squared = 0.967 (Adjusted R Squared = 0.952)

**Table-S2:** Two ways ANOVA of the relations between pH, and temperature in each storage period in related to Phycocyanin of *A. platensis* yield as mg/g dry weight.

Tests of Between-Subjects Effects							

Dependent Variable:dryweight							

Storageperiod	Source	Type III Sum of Squares	df	Mean Square	F	p-value	Partial Eta Squared
3	Corrected Model	0.032^a^	8	0.004	51.048	0.001	0.958
	Intercept	2.478	1	2.478	3.186E4	0.001	0.999
	pH	0.014	2	0.007	90.333	0.001	0.909
	Temp	0.014	2	0.007	87.190	0.001	0.906
	pH *Temp	0.004	4	0.001	13.333	0.001	0.748
	Error	0.001	18	7.778E-5			
	Total	2.511	27				
	Corrected Total	0.033	26				
10	Corrected Model	0.032^b^	8	0.004	62.882	0.001	0.965
	Intercept	2.242	1	2.242	3.560E4	0.001	0.999
	pH	0.015	2	0.007	116.529	0.001	0.928
	Temp	0.014	2	0.007	110.529	0.001	0.925
	pH *Temp	0.003	4	0.001	12.235	0.001	0.731
	Error	0.001	18	6.296E-5			
	Total	2.275	27				
	Corrected Total	0.033	26				
17	Corrected Model	0.034^c^	8	0.004	54.440	0.001	0.960
	Intercept	1.899	1	1.899	2.441E4	0.001	0.999
	pH	0.015	2	0.008	99.190	0.001	0.917
	Temp	0.017	2	0.008	108.048	0.001	0.923
	pH *Temp	0.002	4	0.000	5.262	0.005	0.539
	Error	0.001	18	7.778E-5			
	Total	1.934	27				
	Corrected Total	0.035	26				

a. R Squared = 0.958 (Adjusted R Squared = 0.939) b. R Squared = 0.965 (Adjusted R Squared = 0.950) c. R Squared = 0.960 (Adjusted R Squared = 0.943)

### Growth performance of broiler chicks

Table-2 shows the outcomes of broiler chick growth performance metrics as influenced by PC consumption utilizing three experimental diets control, T1 (1 g) of PC/kg diet and T2 (2 g) of PC/kg diet. At 7 days, there was a significant increase in BW and weight gain in the T2 group compared to the other groups. On the other hand, the consumption of diet significantly decreased in the T2 and T1 groups compared to the control. FCR in the T2 and T1 groups was significantly improved over this time. Throughout day 35, the best weight gain, BW, FCR, and FI results were achieved in broiler chicks in the T2 group. T1 and T2 values were considerably higher than those of the control group. BW and BWG increased linearly (p = 0.05) throughout the feeding period in broilers fed 2 g/kg phycocyanin-supplemented diets. Different phycocyanin levels significantly increased BWG but did not affect total FI. Finally, dietary *A. platensis* phycocyanin had a favorable effect on the ultimate BW, FI, BWG, and FCR of broiler chickens. The improved health of birds may be the reason for this change in expansion characteristics. Its positive effects on intestinal microbiota and increased activity of enzymes for digestion resulted in complete absorption of dry matter and nitrogen in the intestinal tract [32]. It has been shown that ApPC increases the activity of antioxidant enzymes. It enhances apparent metabolizable energy digestibility, protein synthesis, and amino acid nutritional digestibility. In addition, it reduces harmful bacteria [33]. BW, average daily growth, and FCR of rabbits were enhanced by the addition of PC at 50, 100, and 150 mg/kg of diet, which develops in hot environments [34]. Moreover, when *A. platensis* was added to the diets of broilers and weaned piglets, their FCR decreased but their BW and BWG increased [35]. Mirzaie *et al*. [36] discovered that increasing *Spirulina* in diets by 2, 1, or 0.5% had no impact on BW, FI, FCR, or average daily BWG of broilers raised at high temperatures. In addition, earlier research has shown that incorporating *Spirulina* has no appreciable impact on the execution measures of broilers.

**Table 2:** Growth performance as affected using phycocyanin of broiler chickens (means ± SE).

Growth performance	Control	T_1_	T_2_	p-value
Initial body weight (g) at 1 day	72 ± 1.0^a^	72 ± 0.18^a^	72 ± 0.18^a^	0.2
Days 7				
BW	114.97 ± 0.18^b^	115.86 ± 0.43^b^	117.95 ± 0.43^a^	0.001
BWG	42.95 ± 0.36^b^	43.86 ± 0.38^b^	45.95 ± 0.48^a^	0.001
FI (g)	343.68 ± 4.23^b^	290.31 ± 2.08^a^	286.73 ± 1.32^a^	0.001
FCR	8.00^a^	6.61^b^	6.24^b^	0.001
Days 14				
BW	323.13 ± 0.32^b^	326.99 ± 0.64^b^	344.71 ± 1.18^a^	0.001
BWG	208.15 ± 3.48^b^	211.12 ± 0.84^b^	226.76 ± 1.21^a^	0.001
FI (g)	638.94 ± 12.06^a^	589.47 ± 2.98^b^	565 ± 6.24^b^	0.001
FCR	3.069^a^	2.79^b^	2.49^b^	0.001
Days 28				
BW	1239.96 ± 3.60^c^	1269.62 ± 4.80^b^	1364.04 ± 5.02^a^	0.001
BWG	916.83 ± 4.79^c^	942.63 ± 4.81^b^	1019.326 ± 5.24^a^	0.001
FI (g)	954.73 ± 6.76^a^	932.36 ± 12.71^a^	922.73 ± 11.59^a^	0.104
FCR	1.041^a^	0.98^ba^	0.90^a^	0.1
Days 35				
BW	1790.66 ± 9.62^b^	1813.91 ± 8.28^b^	1861.764 ± 5.61^a^	0.001
BWG	497.71 ± 9.27^b^	544.28 ± 11.69^a^	550.69 ± 11.25^a^	0.001
FI (g)	1252.63 ± 5.26^a^	1228.42 ± 6.57^a^	1229.21 ± 9.89^a^	0.001
FCR	2.51^a^	2.25^a^	2.23^a^	0.042

Control, T_1_: 100 mg, T_2_: 200, Means within the same row with different superscripts are significantly different (p < 0.05), FCR=Feed conversion ratio, BW=Body weight, BWG=Body weight gain, FI=Feed Intake

### Blood parameters

Table-3 shows the impact of adding ApPC on serum biochemical parameters in broiler chickens. TPs, Alb, and GL levels were all improved by different APC levels. These changes were the best result for chicks fed an ApPC-supplemented diet containing 2 g/kg ApPC than for chicks fed a diet containing 1 g/kg APC. CHO levels decreased significantly according to different ApPC levels, but ALT and AST levels did not change significantly.

**Table 3:** Blood constituents as affected using phycocyanin in broiler chickens.

Items^2^	Treatments^1^			SE

	Control	T_2_	T_3_	
TP (g/dL)	4.33	4.51	4.69	0.19
Alb (g/dL)	3	3.15	3.22	0.14
GL (g/dL)	1.33^b^	1.36^b^	1.47^a^	0.09
CHO (mg/dL)	93^a^	83d	81^c^	0.32
AST (u/L)	90	88	85	2.82
ALT (u/L)	43	41	38	0.59

Control, T_1_: 100 mg, T_2_: 200, Means within the same row with different superscripts are significantly different (p < 0.05). TP=Total protein, GL=Globulin, Alb=Albumin, CHO=Cholesterol, AST=Aspartate aminotransferase, ALT=Alanine aminotransferase

### Antioxidant-related parameters in liver

Table-4 illustrates how ApPC affects antioxidant enzyme activity in the liver of broiler chickens. All ApPC treatment groups showed an impressive increase in total antioxidant capacity (TAC), CAT, and SOD activities when compared to the control group. Better results have been obtained with increasing supplemented amounts. In addition, hepatic MDA decreased in those given diets supplemented with SPC at 1 and 2 g/kg. GPx, CAT, and SOD comprise the antioxidative enzyme system, which is the first line of defense against inflammation. Astaxanthin scavenges reactive oxygen close to the membrane surface through its polar ring, which raises SOD levels and lowers MDA. Furthermore, it prevents the polyene chain’s radical processes from penetrating the membrane [37]. Equilibrium between the antioxidant system and ROS can be improved by changing the activity of the enzyme mentioned above. An enzyme called SOD protects cells from ROS [38]. In this study, enhanced SOD activity reduced MDA concentration in the plasma of chickens treated with PC. This may be linked to increased CAT and SOD activity, which consume hydroperoxides and lipid peroxides. Broiler chicks fed control (ApPC 0) had lower CAT and SOD activity in their plasma, indicating that heat stress harmed their ability to scavenge free radicals. It is well known that heat stress limits SOD and CAT synthesis and increases MDA and oxidative stress [39]. Therefore, lipid peroxidation is associated with high MDA levels. These findings concur with the previous studies on antioxidant factors, which have found that astaxanthin treatment in animals reduces MDA risk factors [40]. The results of this study demonstrate that PC has strong antioxidant properties, inhibiting CAT and SOD production to prevent the oxidation of fatty acids. The oxidation of extended meat preservation, which is the primary determinant of the quality of meat, helps to explain the aforementioned findings. Increased amounts of polyunsaturated fatty acids make broiler meat more susceptible to oxidative rancidity.

**Table 4:** Antioxidant enzyme activity and lipid peroxidation in liver as affected using phycocyanin in broiler chickens.

Item	Treatments^1^			Sig.

	Control	T_1_	T_2_	
GSH (mmol/g. tissue)	1.73^b^	2.06^ab^	2.25^a^	NS
MDA (mmol/g. tissue)	197^a^	160.7^b^	93^c^	*
TAC (mmol/L)	1.31^b^	1.63^ab^	1.85^a^	*
CAT (u/g, tissue)	2.90^b^	3.42^a^	3.62^a^	*
SOD (u/g. tissue)	157.5^b^	205.2^ab^	213.6^a^	*

Control, T_1_: 100 mg, T_2_: 200, Means within the same row with different superscripts are significantly different (p < 0.05), TAC=Total antioxidant capacity, CAT=Catalase, SOD=Superoxide dismutase, GSH=Glutathione, MDA=Malondialdehyde

### Impact of phycoyanin on amino acid profiles in broiler meat

**Figure-5:**
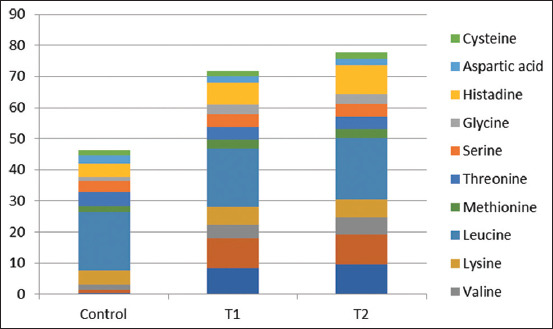
Amino acids contents in meat of broilers chickens which fed on different levels of phycocyanin.

The amino acid content of the meat tissue of broiler chickens fed varying amounts of phycocyanine is summarized in Table-5 and Figure-5. This shows how dietary PC affects the amino acid composition of meat. The concentrations of vital amino acids, alanine, arginine, valine, glycine, and histidine were higher in broilers treated with 1 g ApPC or 2 g APC compared to control birds. Compared to the control, the levels of other amino acids in all experimental groups remained statistically unchanged. Broiler hens fed ApPC 1 g and ApPC 2 g exhibited significantly higher levels of critical amino acids (alanine, arginine, valine, glycine, and histidine acid) in their flesh compared to control hens due to their higher amounts of essential amino acids and TPs. In addition, the presence of strong antioxidants in SPC may contribute to the suppression of amino acid oxidation, which can result from the breakdown of proteins [41]. According to the results of the amino acid profile of the meat, ApPC significantly increased the levels of important amino acids in broiler meat compared to control.

**Table 5:** Profiles of amino acids in meat of broilers chickens fed different levels of phycocyanin.

Amino acids	Control	T_1_	T_2_
Alanine	0.5^b^	8.3^a^	9.5^a^
Arginine	0.9^b^	9.6^a^	9.7^a^
Valine	1.6^a^	4.5^a^	5.5^a^
Lysine	4.7	5.6	5.8
Leucine	18.7	18.9	19.7
Methionine	1.9	2.7	2.9
Threonine	4.5	4.1	4.1
Serine	3.6	4.11	4.11
Glycine	1.2^b^	3.07^a^	3.05^a^
Histadine	4.5^c^	7.1^b^	9.3^a^
Aspartic acid	2.5	2.0	2.02
Cysteine	1.8	1.9	2.01

Control, T_1_: 100 mg, T_2_: 200, Means within the same row with different superscripts are significantly different (p < 0.05)

### Influence of nutritional phycoyanin on fatty acid profiles of broiler meat

**Figure-6:**
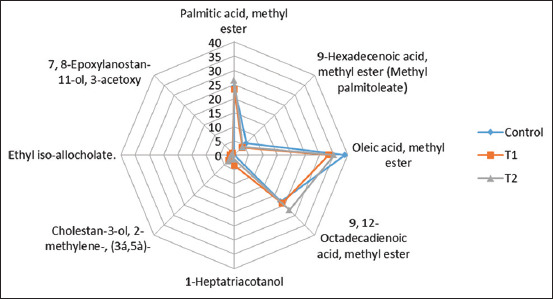
Radar profiles of fatty acid in meat of broilers chickens which fed of different levels of phycocyanin.

The most prevalent FAME was oleic acid (omega-9), with concentrations of 39.13, 33.15, and 37.24 in control, T1, and T2, respectively. According to Milićević *et al*. [42], oleic acid has the highest concentrations in human cells, vegetables, and chicken meat (42.85%). Omega-9 oleic acids are incorporated into the phospholipids found in cell membranes, which are essential for proper membrane fluidity and have anticancer properties. Palmitic acid methyl ester, a saturated fatty acid, is the second most common fatty acid in chicken. According to Dhayabaran and Thangarathinam [43], palmitic acid has antioxidant, hypocholesterolemic, nematicide, pesticide, lubricant, and antiandrogenic properties. The interaction between palmitic acid and stearic acid decreases cardiovascular illnesses [44]. According to Chandrasekaran *et al*. [45], the methyl ester of tetradecanoic acid has antifungal and antibacterial properties. Elaiyaraja and Chandramohan [46] reported that heptadecanoic acid, 16-methyl-, methyl ester (methyl isostearate), is employed to combat skin cancer protein formation (Table-6 and Figure-6).

**Table 6:** Profiles of fatty acid content in broiler chicken meat fed different levels of phycocyanin.

Compound name	Sample Name		

	Control	T_1_	T_2_
Palmitic acid, methyl ester	24.17	23.32	26.48
9-Hexadecenoic acid, methyl ester (Methyl palmitoleate)	4.98	3.77	4.28
Oleic acid, methyl ester	39.13	33.15	37.24
9-12,-Octadecadienoic acid, methyl ester	23.13	23.88	27.39
1-Heptatriacotanol	-	3.50	3.81
Cholestan-3-ol, 2-methylene-, (5à ,3á)-	-	2.61	3.82
Ethyl iso-allocholate	1.77	1.65	2.01
7-8,-Epoxylanostan-11-ol, 3-acetoxy	-	1.03	1.71
17-Pentatriacontene	1.11	1.52	1.90
Oleic acid, 3-(octadecyloxy) propyl este.	1.75	2.59	2.91

T_1_: Control, T_2_: 100 mg, T3: 200, Means within the same row with different superscripts are significantly different (p < 0.05)

### The correlation matrix heat map

**Figure-7:**
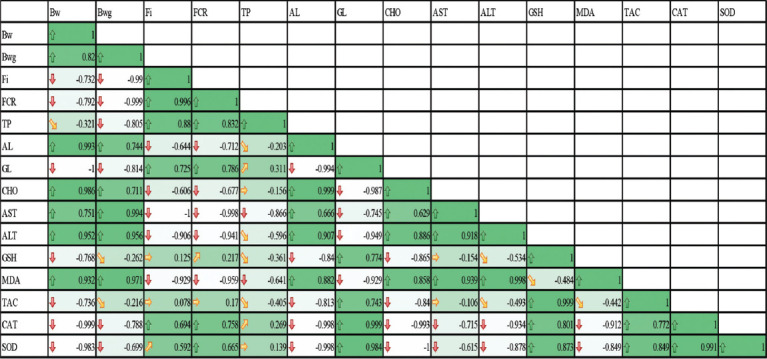
The correlation matrix heatmap displays the values of the Pearson correlation coefficient of measured parameters growth performance (body weight, body weight gain, feed intake, and feed conversion ratio), blood parameters (total protein, albumin, globulin, cholesterol, aspartate aminotransferase, and alanine aminotransferase), and liver antioxidant enzyme activity (glutathione, malondialdehyde, total antioxidant capacity, catalase, and superoxide dismutase).

The correlation matrix heat map clears the values of the Pearson correlation coefficient for the studied parameters, the positive values in green, and the negative values in white. It ranges from 1 to 1, where (1) indicates a perfect positive linear relationship between variables, 1 indicates a negative linear association between variables, and (0) indicates no relationship between measured variables. Growth performance (BW, BWG, FI, and FCR), blood parameters (TP, Alp, GL, CHO, AST, and ALT), and liver antioxidant enzyme activity (GSH, MDA, TAC, CAT, and SOD) were measured. The Pearson’s correlation coefficient analysis and heat map analysis of measured parameters after treatments are shown in Figure-7. Pearson’s correlation coefficient is useful for investigating the inter-correlation between the measured parameters. A strong positive correlation was observed between BWG, Alb, CHO, ALT, and MDA, and the same results were obtained with BWG. Feed intake was positively correlated with FCR, TP, and GL. MDA content was positively correlated with BW, BWG, Alb, AST, ALT, and GSH.

## Conclusion

Incorporating ApPC into broiler chicken feeds can enhance efficiency by increasing final BWG and BW and decreasing FCR without impacting the overall FI. Dietary APC increased serum Alb and TPs and total GL levels. It enhances antioxidant activity as evidenced by increased CAT, TAC, and SOD activities. MDA levels were reduced by ApPC supplementation. As a result, ApPC can be utilized as an alternative natural growth stimulant, antioxidant, and feed supplement in broilers.

## Data Availability Statement

All data generated during the study are included in the manuscript.

## Authors’ Contributions

NME: Conceptualization, data analysis, methodology, and writing. RAH: Conceptualization, data analysis, writing, reviewing, and editing. AAS: Resources and validation. TMA: Resources and validation. TJK: Resources and validation. GAE: Data analysis and methodology. All authors have read, reviewed, and approved the final manuscript.
